# CRISPR RNA binding and DNA target recognition by purified Cascade complexes from *Escherichia coli*

**DOI:** 10.1093/nar/gku1285

**Published:** 2014-12-08

**Authors:** Natalia Beloglazova, Konstantin Kuznedelov, Robert Flick, Kirill A. Datsenko, Greg Brown, Ana Popovic, Sofia Lemak, Ekaterina Semenova, Konstantin Severinov, Alexander F. Yakunin

**Affiliations:** 1 Department of Chemical Engineering and Applied Chemistry, University of Toronto, Toronto, Ontario, M5S 3E5, Canada; 2 Waksman Institute of Microbiology, Rutgers, the State University of New Jersey, Piscataway, NJ 08854, USA; 3 Department of Biological Sciences, Purdue University, West Lafayette, IN 47907, USA; 4 Institute of Molecular Genetics, Russian Academy of Sciences, Moscow 123182, Russia

## Abstract

Clustered regularly interspaced short palindromic repeats (CRISPRs) and their associated Cas proteins comprise a prokaryotic RNA-guided adaptive immune system that interferes with mobile genetic elements, such as plasmids and phages. The type I-E CRISPR interference complex Cascade from *Escherichia coli* is composed of five different Cas proteins and a 61-nt-long guide RNA (crRNA). crRNAs contain a unique 32-nt spacer flanked by a repeat-derived 5′ handle (8 nt) and a 3′ handle (21 nt). The spacer part of crRNA directs Cascade to DNA targets. Here, we show that the *E. coli* Cascade can be expressed and purified from cells lacking crRNAs and loaded *in vitro* with synthetic crRNAs, which direct it to targets complementary to crRNA spacer. The deletion of even one nucleotide from the crRNA 5′ handle disrupted its binding to Cascade and target DNA recognition. In contrast, crRNA variants with just a single nucleotide downstream of the spacer part bound Cascade and the resulting ribonucleotide complex containing a 41-nt-long crRNA specifically recognized DNA targets. Thus, the *E. coli* Cascade-crRNA system exhibits significant flexibility suggesting that this complex can be engineered for applications in genome editing and opening the way for incorporation of site-specific labels in crRNA.

## INTRODUCTION

The Clustered Regularly Interspaced Short Palindromic Repeats (CRISPR) and CRISPR-associated (Cas) proteins form an RNA-guided microbial immune system (CRISPR-Cas), which provides adaptive and inheritable immunity to the host against invading viruses and plasmids (1–4). CRISPR interference is based on short fragments (25–50 nucleotides (nt)) of foreign DNA (protospacers) incorporated into the host chromosome as spacers separated by identical repeats of similar length. Non-coding DNA loci containing repeats and spacers are referred to as CRISPR cassettes or arrays and form a ‘molecular memory’ of prior encounters with mobile genetic elements. The CRISPR cassettes are transcribed and then processed to generate short guide RNAs (crRNAs) containing individual spacers flanked with repeat fragments. The crRNAs form complexes with various Cas proteins and direct Cas nucleases toward target DNAs containing sequences matching crRNA spacers (5–9).

**Figure 1.**
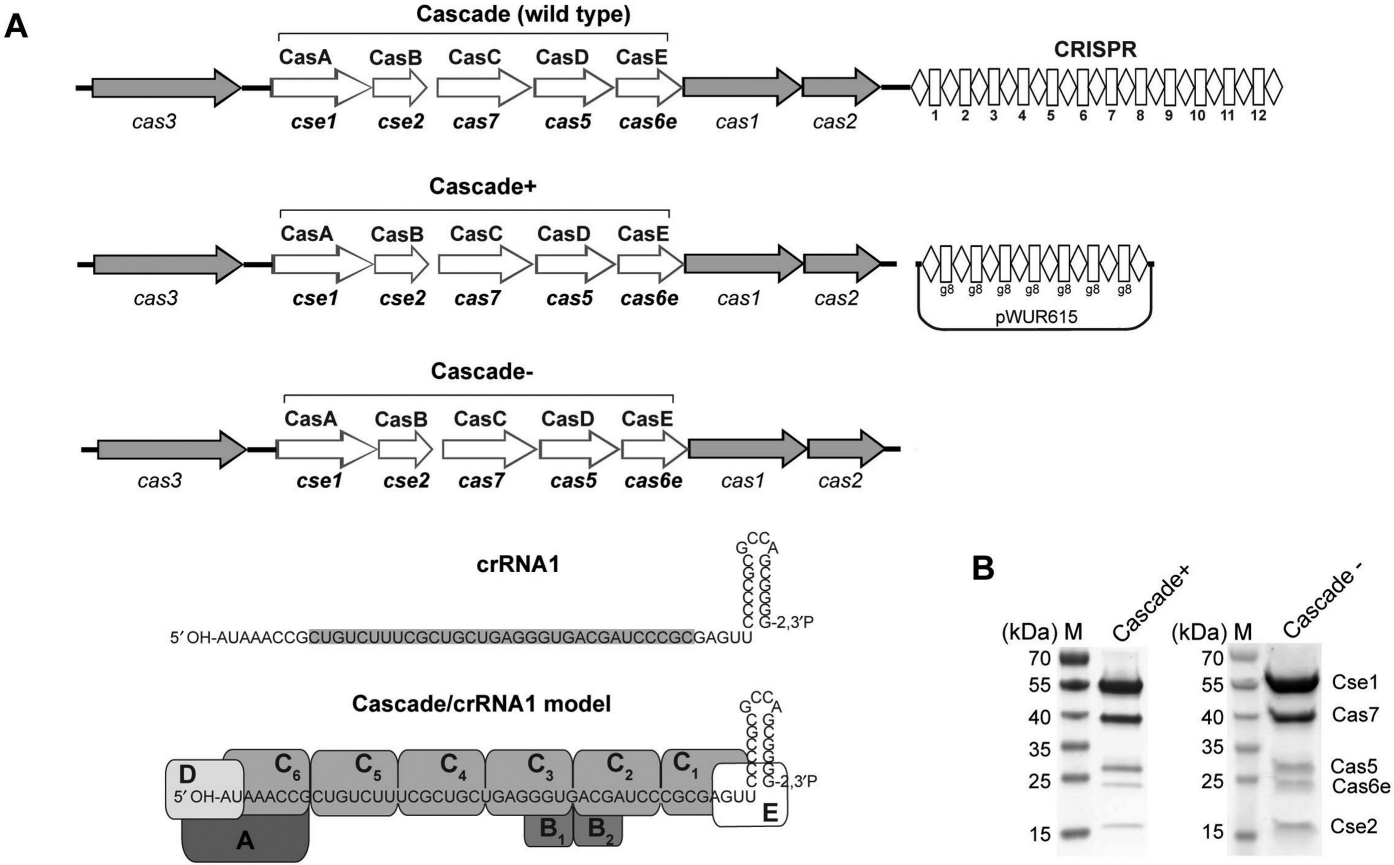
Purification of the *E. coli* Cascade complexes. (**A**) Schematic diagrams of the *E. coli* K12 CRISPR-Cas system (top), a variant used to produce Cascade^+^ loaded with g8 crRNA (middle) and Cascade^−^. Mature g8 CRISPR RNA (crRNA1) and its complex with Cascade are also schematically shown. (**B**) Coomassie-blue staining of SDS-PAGE gel showing the purified *E. coli* Cascade complexes produced with an N-terminal 6His-tag in the presence of g8 crRNA (Cascade^+^) or without crRNA expression (Cascade^−^) in *E. coli* cells with a deleted CRISPR locus. The Cascade samples (7.5 μg protein, after affinity purification) were analyzed on a 4–20% SDS-polyacrylamide gel. Lane M shows molecular weight markers, and the Cascade subunits are indicated.

The CRISPR-Cas systems have been organized into three major groups (types I–III) based on the presence of signature Cas proteins: Cas3 (type I), Cas9 (type II) and Cas10 (type III) (1,2,10). The most prevalent CRISPR type I system is further divided into six subtypes (I-A, I-B, I-C, I-D, I-E and I-F) depending on the presence of subtype-specific genes (2). Both type I and III systems involve the Cas6 family of endoribonucleases which cleave the repeat sequences of the crRNA precursor to generate short crRNAs with 8 nt of repeat sequence at their 5′ ends (5′ handle) (11,12). DNA target recognition in both types is carried out by a multi-subunit ribonucleoprotein complex, which is called Cascade in *Escherichia coli* (*C*RISPR-*as*sociated *c*omplex for *a*ntiviral *de*fense, type I-E), CSM in type III-A systems or CMR in type III-B systems (7). The *E. coli* Cascade-crRNA complex (405 kDa) is composed of five proteins (11 subunits): one Cse1 (CasA), two Cse2 (CasB), six Cas7 (CasC), one Cas5 (CasD), one Cas6e (CasE) and a 61-nt crRNA with a 5′-hydroxyl and a 2′, 3′-cyclic phosphate at the ends (Figure 1A) (7,13). The mature *E. coli* crRNA contains a 32-nt spacer flanked by a repeat-derived 5′ handle (8 nt) and a 3′ handle (21 nt) which forms a hairpin (Figure 1A) (13).

For efficient DNA target binding by Cascade, two elements are essential: a PAM (protospacer adjacent motif) and a seed sequence. PAMs are conserved 2–5 nt sequences located immediately upstream or downstream of protospacers, whereas the seed is composed of the first 8–10 nt of the crRNA spacer adjacent to PAM and must be strictly complementary to the target sequence (except for the sixth nucleotide position) (14–17). The PAM motifs play an important role in the CRISPR adaptation and interference steps in both type I and type II systems, but PAMs have not been identified in type III systems (18). The present model of target recognition suggests that Cascade first recognizes the PAM motif through short-lived protein:DNA interactions followed by the hybridization of the crRNA:DNA seed sequences and, eventually, of the entire spacer-protospacer region (14). The *E. coli* Cascade recognizes double-stranded (ds) DNA targets. Following PAM recognition by Cse1 and base pairing between the crRNA and complementary target DNA strand an R-loop with displaced non-complementary target DNA strand is formed (13). The formation of the R-loop might be stabilized by the two Cse2 subunits and is followed by the recruitment of a Cas3 nuclease-helicase, which cleaves the R-loop and degrades the target DNA (19–22). The cryo-electron microscopy structure of the *E. coli* Cascade revealed a seahorse-shaped configuration with the 3′ and 5′ crRNA handles fixed at the opposite ends of the complex and the crRNA spacer aligned along the helical backbone of six Cas7 (CasC) proteins (19). This model has been confirmed by recent crystal structures of the *E. coli* Cascade, which revealed that the crRNA guide sequence is exposed along a helical assembly of six interwoven Cas7 subunits, whereas the 3′ and 5′ ends of crRNA are anchored by Cas6 and Cas5, respectively (23–25). Electron microscopy structures of the CSM and CMR complexes from several organisms also showed an elongated design with a multi-copy backbone composed of Csm3 or Cmr4, which is similar to the Cascade backbone (26–28).

Biochemical studies of purified type I and III CRISPR effector complexes from various organisms have demonstrated the presence of bound crRNAs, most of which contained a single spacer flanked by fragments of neighboring repeats. Almost 80% of cloned crRNAs extracted from the *E. coli* Cascade started with the last 8 nt of the repeat sequence (AUAAACCG; 5′ handle) followed by a complete spacer sequence (32 or 33 nt) and a variable-length 3′ handle originating from the next repeat (7). Sequencing of cloned crRNAs showed that most of the 3′ handles varied from 8 to 19 nt, while some crRNAs had no 3′ handle (7). However, the electrospray ionisation mass spectrometry (ESI-MS) analysis of crRNA extracted from the *E. coli* Cascade, co-expressed with an engineered CRISPR cassette containing seven identical spacers, revealed a single 61 nt crRNA, which must have been produced from a single cleavage by the Cas6e (CasE) endoribonuclease within the repeat at the hairpin base (8 nt upstream of the spacer) (13). Thus, the present model of the mature *E. coli* crRNA proposes a 61-nt-long sequence, which includes an 8 nt 5′ handle, a 32 nt spacer and a 21 nt 3′ handle with a hairpin. Interestingly, the 8-nt-long 5′ handles were also identified in most crRNAs from *Staphylococcus epidermidis*, *Pyrococcus furiosus*, *Sulfolobussolfataricus* and *Thermus thermophilus* suggesting that they are a general feature of crRNAs (5,27,29–31). However, the 3′ ends of sequenced crRNAs were highly variable, and crRNAs of different sizes were found to bind to the same effector complex. The CMR complexes from *P. furiosus* and *T. thermophilus* and the *S. epidermidis* CSM complex showed the presence of two major crRNA species with different 3′ ends: 39/40 nt and 45/46 nt, whereas the CSM crRNA pool from *S. solfataricus* comprised one major type of crRNA (∼50 nt) with the average 3′ handle containing 3 nt (5,12,26,27). Biochemical analysis indicated the presence of 5′ and 3′ hydroxyl groups in the crRNA extracted from the *S. solfataricus* CMR, whereas the crRNA from *E. coli*, *T. thermophilus* and *P. furiosus* effector complexes was phosphorylated at the 3′ end (2′, 3′-cyclic phosphate) (5,13,27,31). In *P. aeruginosa*, the endoribonuclease Csy4 cleaves pre-crRNA with the formation of products containing 5′-hydroxyl and 3′-phosphate (or 2′-3′ cyclic phosphate) groups (32). Furthermore, analysis of the crRNAs bound to various CRISPR effector complexes showed a broad range of spacer lengths with the *S. solfataricus* Cascade and CSM complexes accommodating spacers from 34 to 44 nt (26,33). Finally, several studies have identified the presence of significant amounts (up to 15%) of non-CRISPR RNAs in various CRISPR effector complexes including the *E. coli* Cascade and the *S. solfataricus* CSM, CMR and Cascade complexes (7,26,30,33,34). For example, the *S. solfataricus* CSM complex has been found to contain high levels of the non-CRISPR RNA ncRNA60, whose synthetic precursor can be cleaved by the purified *S. solfataricus* Cas6–3 generating a mature ncRNA60 with a 5′ sequence resembling the crRNA 5′ sequence (34). Thus, CRISPR-associated effector complexes exhibit significant structural and functional flexibility including processing and binding of non-CRISPR RNAs.

Here, we show that a functionally active Cascade complex can be expressed and purified from *E. coli* cells lacking CRISPR cassettes and, therefore, devoid of crRNAs. Cascade purified from such cells can be loaded *in vitro* with synthetic crRNAs, which direct the resulting complex to complementary dsDNA targets resulting in the formation of an R-loop. *In vitro* Cascade binding and DNA target recognition experiments using synthetic crRNA derivatives demonstrated that all eight nucleotides of the 5′ handle are required for these activities. In contrast, a single nucleotide, with any base, downstream of the spacer part of crRNA is sufficient for Cascade binding and DNA target recognition.

## MATERIALS AND METHODS

### Gene cloning and protein purification

For recombinant expression and purification of the *E. coli* Cascade, the Cascade operon (*casABCDE*) was subcloned from the plasmid pWUR400 (7) into a T7 RNA polymerase-based protein expression vector p15Tv-L using ligation-independent cloning, which adds an N-terminal 6His-tag to the Cse1 (CasA) protein. The recombinant plasmid was sequenced to verify the presence of all Cascade genes. The Cascade overexpression plasmid was transformed into the *E. coli* BL21(DE3) strain or into the modified BL21(DE3) strain KD418 with a deleted CRISPR cassette (genome position 1002803–1003730). The deletion of the CRISPR cassette was performed using the previously described Red recombinase protocol (35). For co-expression of Cascade proteins with crRNA, the Cascade overexpressing strain was co-transformed with the pWUR615 plasmid containing seven g8 spacers (32 nt each) complementary to the phage M13 gene 8 (14).

### Protein expression and purification

The Cascade complexes were overexpressed in the *E. coli* BL21 (DE3) Star strain with a deleted CRISPR1 cassette (KD418). For crRNA co-expression (for Cascade^+^), this strain was co-transformed with the plasmid pWUR615 expressing seven g8 spacers complementary to the phage M13 gene 8 (14). Cells were grown in TB media to OD_600_ = 0.8 at 37°C, induced with 0.3 mM Isopropyl β-D-1-thiogalactopyranoside (IPTG) overnight at 17°C and purified using a nickel-affinity column. The buffers for affinity purification contained: 50 mM HEPES-K (pH 7.5), 300 mM NaCl, 5% (v/v) glycerol and 0–150 mM imidazole. The eluted protein complex was further purified using a Superdex 200 HiLoad 16/60 column (Amersham Biosciences) equilibrated by 20 mM HEPES-K buffer (pH 7.5) containing 150 mM NaCl. The purified complex was analyzed by sodium dodecyl sulphate-polyacrylamide gel electrophoresis (SDS-PAGE), and the presence of Cascade proteins was confirmed using mass spectrometry (MS). Protein concentration was determined by Bradford assay using bovine serum albumin as a reference protein with the conversion to molar Cascade concentrations using the previously reported Cascade stoichiometry (13).

### Mass spectrometry

Purified complexes were analyzed by MS/MS on a Thermo Q-Exactive mass spectrometer equipped with a Nanospray Flex Ion Source, Thermo Scientific Acclaim PepMap RSLC 50 μm x 15 cm, nanoViper C18, 2 μm column and an Easy-nLC 1000 high pressure liquid chromatography system. The mobile phase consisted of two eluants: 0.1% formic acid (Buffer A) and acetonitrile (Buffer B). The conditions used were: 5 min at up to 10% B, 101 min at up to 40% B, 103 min at up to 95% B and 120 min with up to 95% B, with a flow rate of 250 nl/min. Raw data files were converted with MSconvert and analyzed using a custom database in GPM Manager.

For the identification of individual Cas proteins in solution, 10 μg of total protein was first incubated with 1 μg of Trypsin in 50 mM ammonium bicarbonate (pH 8.5), for 1 h at 37°C. After that an additional 1 μg of Trypsin was added and samples were incubated overnight at 37°C. Cleanup of samples was done using Agilent Omix C18 pipette tips as per manufacturer instructions, dried in a Speedvac and re-suspended in 100 μl of 0.1% formic acid. Peptide identification from the SDS-PAGE gels was conducted following a protocol modified from Shevchenko *et al.* (36). Following fixation, gels were washed in MilliQ water for several hours and stained bands were excised and transferred into microcentrifuge tubes. Gel fragments were incubated for 30 min at room temperature in 100 μl of ammonium bicarbonate/acetonitrile (1:1, vol/vol), then 500 μl of acetonitrile was added. After the color disappeared, the solution was removed, gel pieces were covered with 50 μl of trypsin buffer (13 ng/μl trypsin in 10 mM ammonium bicarbonate, pH 8.5, 10% acetonitrile) and left on ice for 30 min. An additional 50 μl of trypsin buffer was added and the samples were kept on ice for 90 min followed by addition of 20 μl of 10 mM ammonium bicarbonate (pH 8.5) and incubation at 37°C overnight. Cleanup and analysis of samples was done as previously described (36).

### Preparation of nucleic acid substrates

The ssDNA and RNA oligonucleotides used in this work (Supplementary Table S1) were purchased from IDT (USA). The oligonucleotides were [^32^P]-labeled at the 5′-end using T4 polynucleotide kinase (PNK, BioLabs) and purified as previously described (37). The synthetic crRNA1 substrate (61 nt) contains a 32-nt spacer sequence from the phage M13 gene g8 flanked by the *E. coli* repeat-derived 5′ and 3′ handles (8 and 21 nt, respectively). The crRNA MS2 (61 nt) has a 32-nt spacer sequence from the phage MS2 gene 7 flanked by the *E. coli* repeat derived 5′ and 3′ handles (as in crRNA1). The synthetic g8 ssDNA (79 nt) contains a 32-nt protospacer complementary to the M13 phage gene 8 (g8) sequence with the ATG PAM flanked by the g8-derived sequences on the 5′ end (25 nt) and 3′ end (22 nt). The MS2 ssDNA (72 nt) contains a 32-nt protospacer, complementary to the MS2 phage gene 7 sequence, with the ATG PAM flanked by the MS2-derived sequences (20 nt each) on the 5′ and 3′ ends. Double-stranded substrates were prepared by incubating 5′-[^32^P]-labeled sense and unlabeled complementary strands at a molar ratio 1:1.5 in 10 mM Tris-HCl (pH 8), for 4 min at 90°C, 10 min at 37°C and for 30 min at room temperature. The synthetic g8 dsDNA (79 bp) was prepared by annealing DNA1 and DNA2, the mutant g8 dsDNA (C1T) using DNA3 and DNA4, the non-target dsDNA using DNA5 and DNA6, and the dsDNA MS2 using DNA7 and DNA8 (Supplementary Table S1).

### Electrophoretic mobility shift assays (EMSAs)

Nucleic acid binding was carried out for 30 min at 30°C or 37°C using 10 nM ss- or ds-5′-[^32^P]-labeled substrates in a 10 μl reaction mixture containing 20 mM Tris-HCl (pH 8) and 20 mM NaCl. Labeled ss- or ds-substrates were incubated in the presence of increasing concentrations of Cascade and the reactions were quenched by the addition of a glycerol loading dye to 10% followed by electrophoresis in 6% native gels and Phosphorimager visualization. The bands of the unbound and bound probes were quantified using ImageQuant software (GE Healthcare). The fraction of bound probe was plotted against the total Cascade concentration, and the data were fitted by non-linear regression analysis to the following equation: Fraction of bound probe = [Cascade]total/(*K*d + [Cascade]total). *K*_d_ values reported are the average of three independent determinations.

### Permanganate probing

Oxidative modification by KMnO_4_ was performed with 5 nM labeled target DNA, 1–2 μg of Cascade complex and 0.5–1 μM crRNA in 10 μl binding buffer (40 mM Tris-HCl, pH 8.0, 50 mM NaCl). Cascade complexes were first incubated with crRNA1 at 37°C for 5 min to allow the formation of a Cascade-crRNA complex. After that the 5′-[^32^P]-labeled g8 dsDNA (79 bp) was added to the Cascade-crRNA complex and incubated at 37°C for 25–30 min. Oxidative modification reactions were initiated by adding KMnO_4_ to a final concentration of 2 mM and incubated for 15 s at 37°C. Reactions were quenched by adding 10 μl of 1% β-mercaptoethanol and 6 μg of calf thymus DNA in 50 μl of 10 mM Tris-HCl (pH 8.5) and extracted with phenol-chloroform (49:1), followed by ethanol precipitation. DNA pellets were dissolved in 100 μl of freshly prepared 1 M piperidine and heated in a boiling water-bath for 10 min. Piperidine was removed by chloroform extraction and DNA was precipitated by ethanol. Pellets were dissolved in a formamide loading dye and analyzed on an 8% denaturing gel. After electrophoresis, gels were fixed in 10% acetic acid, dried and visualized by phosphorimaging.

## RESULTS

### Purification of recombinant crRNA-free Cascade complex from *E. coli*

Previously, recombinant *E. coli* Cascade complexes were purified from cells co-expressing Cas proteins with affinity tags either on Cse2 (CasB), Cas7 (CasC), Cas5 (CasD) or Cas6e (CasE) subunits and individual crRNAs from a compatible plasmid harboring an engineered CRISPR array (7,13). For example, *E. coli* Cascade complexed with g8 crRNA containing a 32-nt spacer sequence from the phage M13 gene 8 was found, when purified, to efficiently and specifically recognize M13 DNA (14,20). We found that using the same setup the *E. coli* Cascade can also be purified using an N-terminal His_6_-tag attached to Cse1 (CasA) (Figure 1B). SDS-PAGE analysis of protein fractions from the Ni^2+^-chelate affinity chromatography columns revealed the presence of all five Cascade components whose identity was confirmed using in-gel trypsin digestion and liquid chromatography-MS (LC-MS) analysis (Supplementary Table S2). This complex was labeled as Cascade^+^ to indicate that it was co-expressed with the g8 crRNA. The purified Cse1-tagged Cascade^+^ shows higher levels of Cse1 compared to the StrepTactin purified Cse2-tagged Cascade (13) suggesting that Cascade^+^ also contains significant amounts of free Cse1. This is an expected result since *cse1* is the first (and therefore more highly expressed) gene in the *cse1-cse2-cas7-cas5-cas6e* (*casABCDE*) operon and its product contains the N-terminal 6His-tag used for complex purification. Size-exclusion chromatography of the Ni^+^-column eluate showed the presence of a major ∼400 kDa peak, which based on the SDS-PAGE and LC-MS (in solution) analyses, contained all five Cascade proteins (Supplementary Figure S1 and Supplementary Table S2).

The same purification protocol was used to obtain Cascade from *E. coli* BL21(DE3) cells lacking a functional chromosomal CRISPR cassette and without the plasmid (pWUR615) from which g8 crRNA is produced (Figure 1A). In this way, a Cascade^−^ preparation, which also contained five Cascade protein components based on SDS-PAGE analysis and had the same subunit composition as Cascade^+^ based on LC-MS analysis (Figure 1B; Supplementary Table S2), was obtained. Compared to the previously described *E. coli* Cascade complex (13), both Cascade^+^ and Cascade^−^ contain higher levels of the Cse1 protein, but similar levels of the other Cascade subunits suggesting that both preparations contain both the full complex and Cse1 which is present in excess. Yet, the results clearly suggest that the recombinant Cascade complex can be purified in the absence of crRNA, as has also been noted previously (7). In addition, recent work on the archaeal type I-A Cascade from *Thermoproteus tenax* indicated that a co-refolded Cascade complex can be reconstituted *in vitro* from the six purified Cascade proteins in the absence of added crRNA (38).

### Binding of the purified Cascade^−^ to RNA and DNA

**Figure 2.**
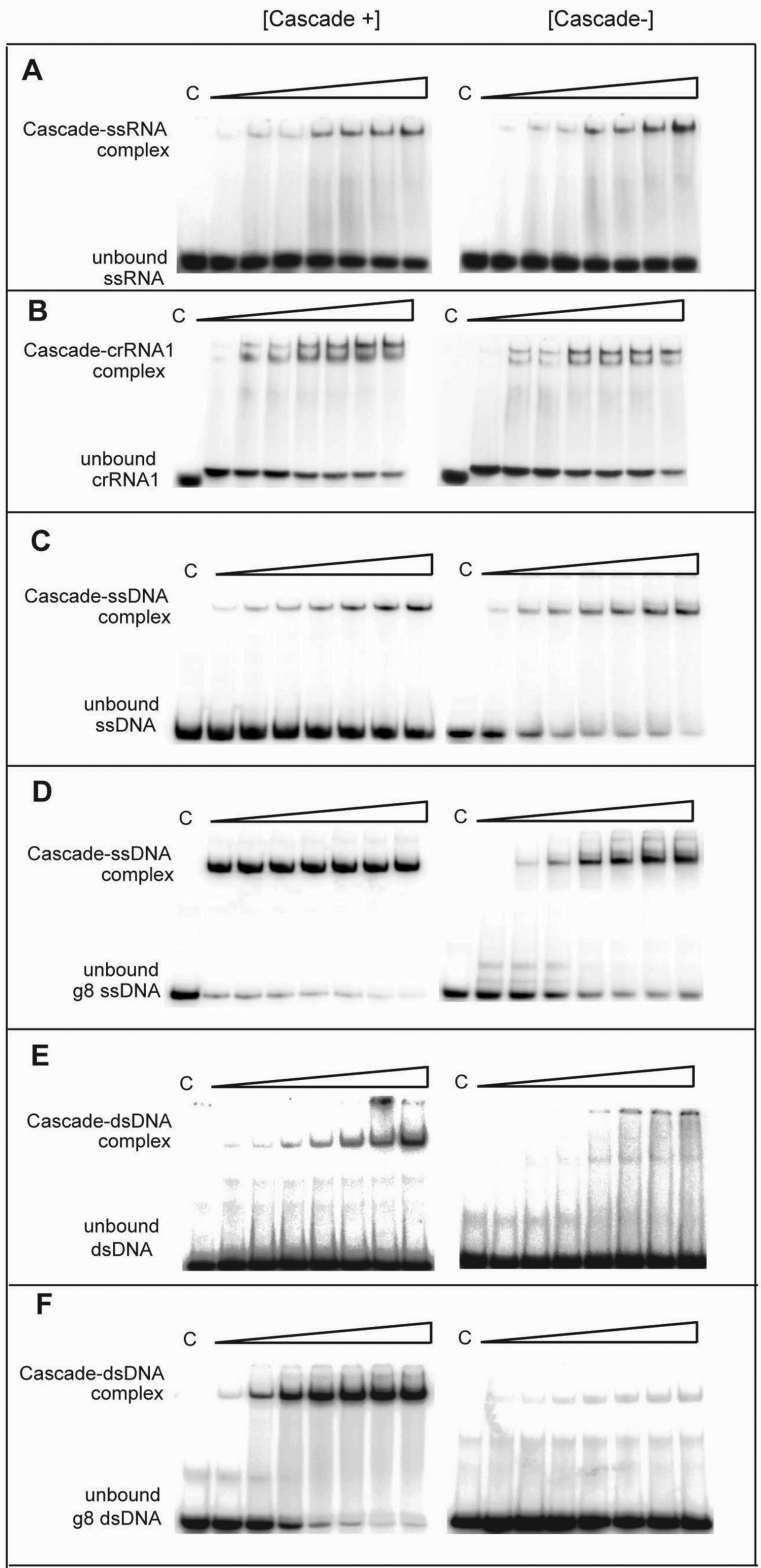
Binding of RNA and DNA by purified Cascade complexes. EMSAs showing binding of the purified Cascade^+^ (left panels) and Cascade^−^ (right panels) to different 5′-[^32^P]-labeled RNAs and DNAs. (**A**) Non-target ssRNA (61 nt); (**B**) target crRNA1 (61 nt); (**C**) non-target ssDNA (80 nt); (**D**) target g8 ssDNA (79 nt); (**E**) non-target dsDNA (80 bp); (**F**) target g8 dsDNA (79 bp). Purified Cascade complexes (0–800 nM) were incubated with the indicated substrates for 30 min at 37°C. Reaction products were resolved by native PAGE (6%) and visualized by phosphorimaging. Lanes C represent assays without protein addition (in all figures).

*E. coli* Cascade purified in the absence of co-expressed crRNA was not characterized biochemically (7). The established paradigm for CRISPR effector-crRNA complexes suggests that after cleavage of pre-crRNA the mature crRNA remains tightly bound to the effector complex during its lifetime (39–41). The presence of up to 15% of non-crRNA in the purified effector complexes produced with co-expression of crRNA was revealed in *E. coli* Cascade and the Cascade-like CSM and CMR systems from *S. solfataricus* (7,26,30,34). We analyzed the binding of purified Cascade^−^ to non-crRNA and crRNA *in vitro*. Mature *E. coli* crRNA (61 nt) includes a 32-nt spacer sequence flanked by a linear 5′ handle (8 nt) and a 3′ handle (21 nt) that forms a hairpin (Figure 1A) (13). According to convention, the 5′ handle nucleotides are labeled with (−), whereas those from the 3′ handle with (+) (23,24). Therefore, the mature *E. coli* crRNA with the full-length handles is referred to as −8/+21. In EMSAs, both Cascade^+^ and Cascade^−^ showed stronger binding to synthetic g8 crRNA (crRNA1) compared to non-CRISPR ssRNA suggesting higher affinity to ‘cognate’ RNA (Figure 2). The formation of double-shifted bands shown in Figure 2B, might be associated with the presence of incomplete Cascade complexes in our preparation. Similar results were obtained using the 3′-[^32^P]-labeled crRNA1 indicating that the presence of phosphate on the crRNA1 5′-end has no effect on binding to Cascade. Both Cascade preparations also bound non-target and target (g8) ssDNA (Figure 2). As expected, Cascade^+^ showed much stronger binding to ssDNA containing the g8 protospacer and functional ATG PAM, whereas Cascade^−^ showed similar binding to both non-target and target ssDNAs (Figure 2). Both Cascade preparations exhibited low binding to the non-target dsDNA, whereas Cascade^+^ showed strong binding to the g8 dsDNA suggesting that it is functionally active. In contrast, Cascade^−^ showed very low binding to the g8 dsDNA (Figure 2), an expected result as it was purified from cells that lacked the source of this or any other crRNA.

### A crRNA-free Cascade loaded with exogenous synthetic crRNA specifically recognizes target DNA

**Figure 3.**
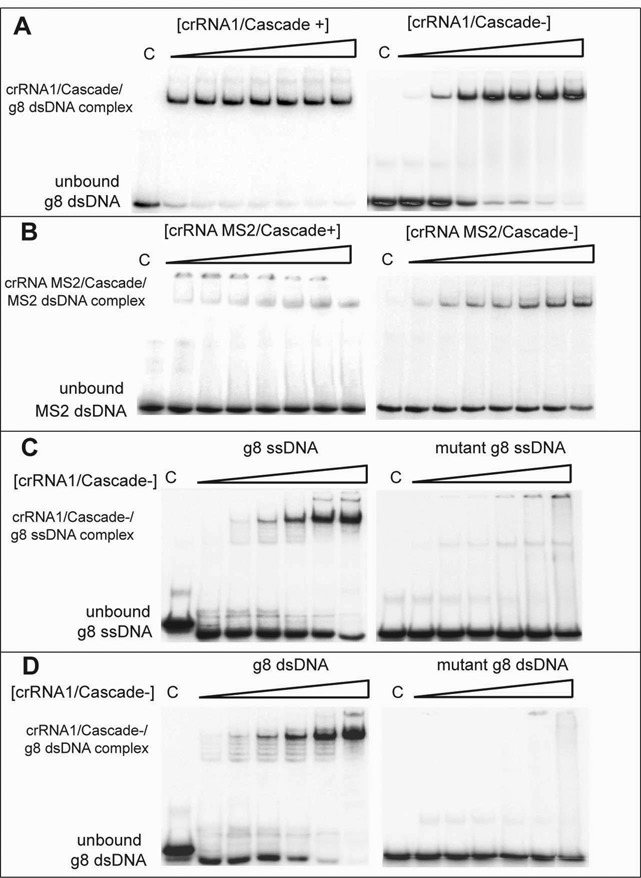
Effect of crRNA loading on Cascade binding to DNA targets. EMSA assays showing DNA binding by the Cascade complexes pre-incubated (loaded) with different crRNAs. (**A** and **B**) Loading of purified Cascades with non-labeled crRNA1 (A) or crRNA MS2 (B) and binding of the loaded Cascade complexes to the corresponding 5′-[^32^P]-labeled target dsDNAs. (**C** and **D**) DNA binding of the crRNA1-loaded Cascade^−^ to the ss- (C) or ds-DNA (D) targets containing the wild-type or mutated (C1T) g8 protospacer. Purified Cascade complexes (0–800 nM) were pre-incubated (10 min at 30°C) with the non-labeled crRNA1 (0.5 μM) followed by the addition of 5′-[^32^P]-labeled g8 dsDNA or MS2 dsDNA (10 nM) and incubated for 30 min at 37°C. Reaction products were resolved by native PAGE (6%) and visualized by phosphorimaging.

Stronger binding of Cascade^−^ to crRNA1 compared to non-crRNA (Figure 2A and B) suggested that it might be possible to load Cascade^−^ with different crRNAs to allow specific recognition of various DNA targets. To verify this hypothesis, we pre-incubated both Cascade^+^ and Cascade^−^ with crRNA1 and analyzed the binding of the resulting complexes to the target g8 dsDNA using EMSA. As expected, pre-incubation of Cascade^+^ with crRNA1 had a small stimulating effect on its binding to the target g8 dsDNA, presumably because a fraction of this Cascade preparation either contains non-crRNA (∼15% according to Jore *et al.* (13)), which is displaced by crRNA1 (Figure 3A), or is crRNA-free. However, pre-incubation of Cascade^−^ with crRNA1 resulted in strong binding of this complex to g8 dsDNA (compare Figures 2F and 3A) implying that the purified Cascade^−^ complex can be loaded with crRNA *in vitro*. Similarly, pre-incubation of both complexes with the MS2 crRNA (contains a spacer matching a sequence from MS2 phage flanked by the *E. coli* 5′ and 3′ handles) induced strong binding of Cascade^−^ to the target MS2 dsDNA, whereas Cascade^+^ showed lower binding, an expected result since most of this Cascade is already loaded with crRNA1 produced inside the cells (Figure 3B). These results suggest that the purified Cascade^−^ can be loaded with any mature crRNA *in vitro*, and that once Cascade forms a stable complex with crRNA, it can not readily be replaced by another crRNA *in vitro*.

For several CRISPR-Cas systems including *E. coli* and *Streptococcus thermophilus*, it has been shown that CRISPR interference was inhibited or even completely eliminated by single mutations in the protospacer seed sequence or in the PAM (escape mutants) (14,42,43). To check if DNA target recognition by the reconstituted Cascade-crRNA1 complex would be sensitive to an escape mutation in the protospacer, we used the g8 ssDNA and g8 dsDNA targets with the C1T substitution in the protospacer sequence (the first nucleotide after the ATG PAM mutated to thymine). This substitution has been shown to allow the M13 phage to escape CRISPR-Cas interference *in vivo* and to strongly decrease Cascade^+^ binding to target DNA *in vitro* (14). As shown in Figure 3C and D, incubation of the crRNA1 loaded Cascade^−^ complex with both the ssDNA and dsDNA mutated g8 targets revealed greatly reduced or no DNA target recognition, in contrast to strong binding to non-mutated targets. Thus, our results imply that purified Cascade^−^ complexes loaded with crRNA *in vitro* can form a functionally active effector-crRNA complex able to recognize and bind the cognate target DNA sequence.

**Figure 4.**
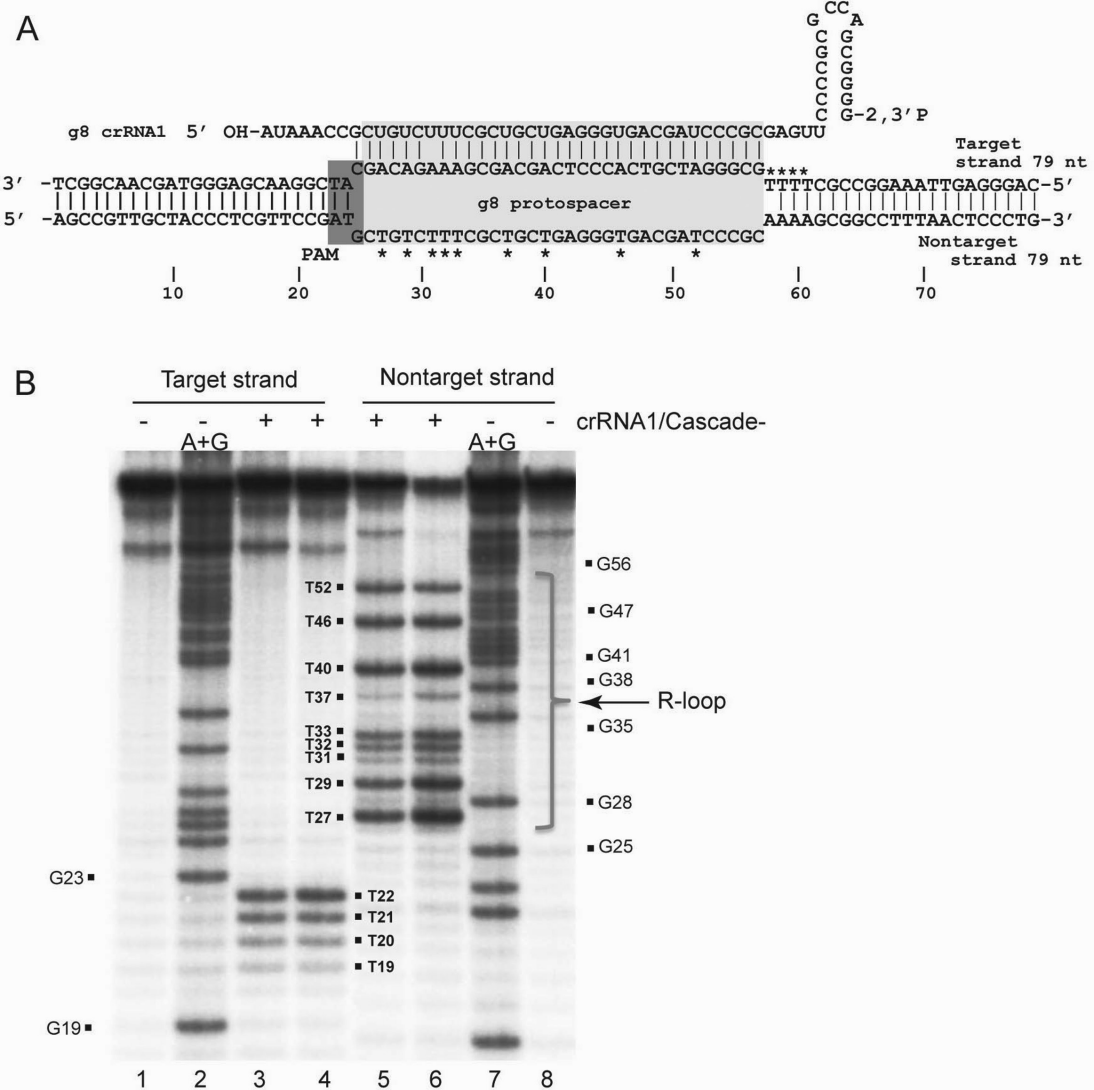
Formation of R-loop by purified Cascade complexes. (**A**) Schematic model of R-loop formation during the binding of the crRNA1-loaded Cascade complex to the target dsDNA (g8 dsDNA). (**B**) Permanganate probing of ssDNA regions in the target (lanes 1–4) and non-target (lanes 5–8) strands of the g8 dsDNA (79 bp) induced by the binding of the crRNA1-loaded Cascade^−^ complex. dsDNA with the 5′-[^32^P]-labeled target or non-target strands (as indicated) was treated with 2 mM KMnO_4_ alone (lanes 3 and 5) or KMnO_4_ with the addition of 5 mM MgSO_4_ (lanes 4 and 6). Lanes 1 and 8, g8 dsDNA treated with KMnO_4_; lanes 2 and 7, (A+G) ladder. The positions of the KMnO_4_-sensitive thymine (T) nucleotides on the target and displaced DNA strands are indicated on the gel and on panel A (by asterisks).

It has been shown that binding of the *E. coli* Cascade-crRNA complex to a target dsDNA induces the formation of an R-loop with the spacer part of the crRNA strand inserted into the duplex DNA: base pairing with one DNA strand, while displacing the other as a ssDNA loop (13,44,45). These R-loops constitute the signal for target DNA degradation by a Cas3 nuclease, and their formation is also dependent on the presence of PAM sequences, which provide the self–non-self discrimination in type I CRISPR-Cas systems (42). To determine if loading of Cascade^−^ with crRNA1 *in vitro* produces a functionally active Cascade-crRNA complex capable of forming an R-loop upon binding to a target DNA, we used a permanganate (KMnO_4_) probing method which is based on selective oxidation of thymines in ssDNA (46). Permanganate probing of the Cascade^−^-crRNA1-g8 dsDNA reaction identified nine thymines located in the central part of g8 protospacer non-target strand as susceptible for permanganate oxidation (Figure 4). In the target DNA strand, the four thymines located just downstream of the g8 protospacer (T22, T21, T20 and T19) were detected with a gradually reducing band intensity suggesting that Cascade binding induced some DNA duplex breathing (a limited DNA duplex opening) immediately downstream of the protospacer (Figure 4). The same pattern of KMnO_4_ sensitivity was observed in reactions with the Cascade^+^ preparation (Supplementary Figure S3). These results imply that the protospacer region of the non-target DNA strand is displaced due to formation of an R-loop by the Cascade^−^-crRNA1 complex. Previous work on the formation of an R-loop by the *E. coli* Cascade was performed at 30°C and revealed that only the first half of the non-target strand of the protospacer was exposed in the Cascade-DNA complex and the second part of the protospacer was shielded (13). In our experiments (performed at 37°C), the entire protospacer area of the non-target g8 DNA strand and the four nucleotides downstream of the protospacer on the target DNA strand were accessible for permanganate oxidation (Figure 4). Overall, our results indicate that the Cascade^−^ complex can be loaded *in vitro* with a mature crRNA, which promotes its specific binding to the complementary target dsDNA with the formation of an R-loop.

### The role of 5′ and 3′ handles of crRNA in binding to Cascade and target DNA

**Figure 5.**
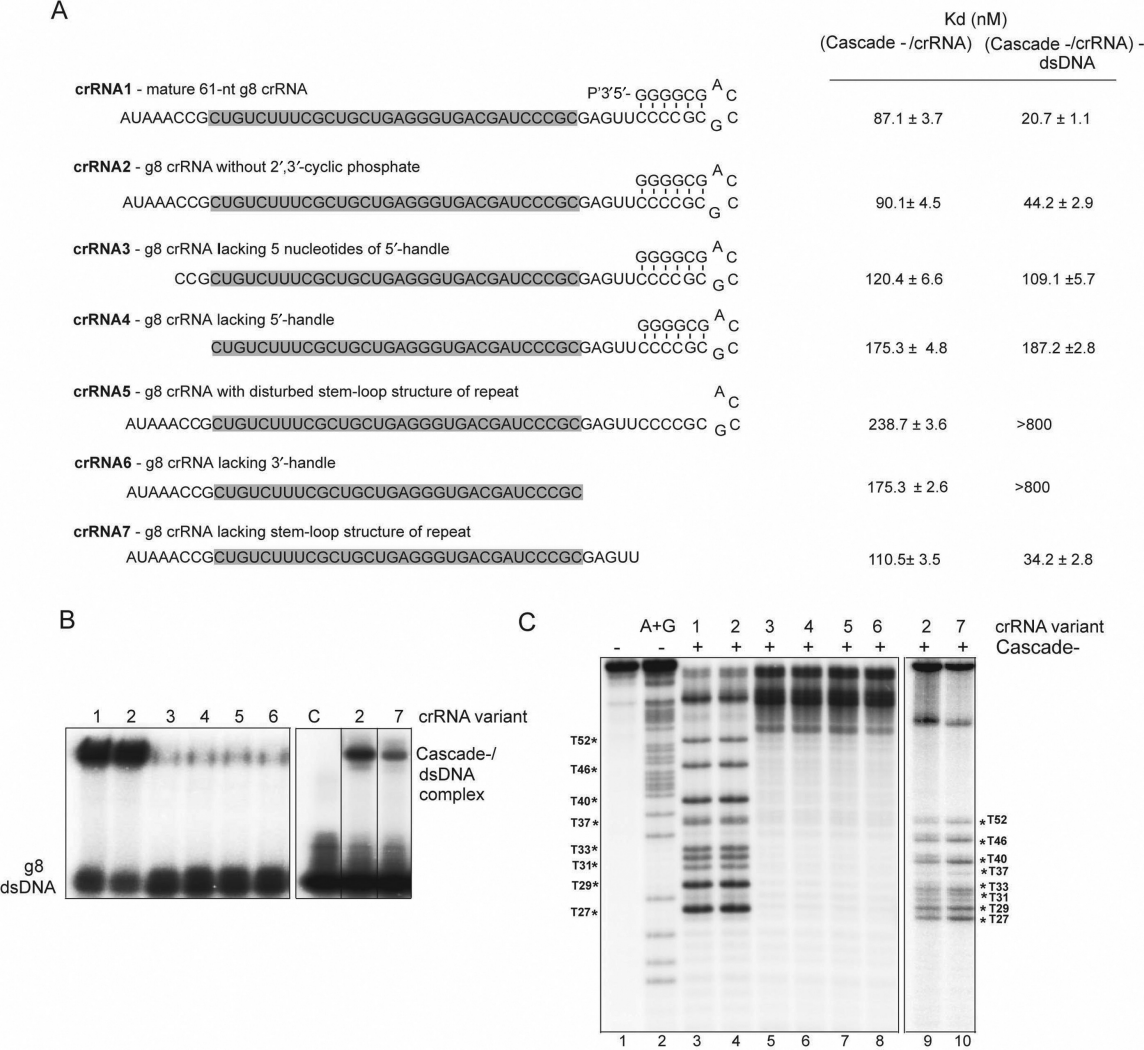
Effect of crRNA truncation on Cascade binding and target DNA recognition. (**A**) Nucleotide sequences of truncated crRNA1 variants used in this work and the dissociation constants (*K*_d_) for binding of these crRNAs to Cascade^−^ (left column) or of the corresponding Cascade^−^/crRNA complexes to g8 dsDNA (79 bp). The dissociation constants were determined by EMSAs using three independent experiments (the corresponding gels are shown in Supplementary Figure S2). The spacer sequences of crRNAs are shaded. (**B**) Binding of the 5′-[^32^P]-labeled g8 dsDNA by the Cascade^−^ complexes loaded with indicated crRNA1 variants (crRNA1 to crRNA7). (**C**) Analysis of R-loop formation by the Cascade/crRNA/g8 dsDNA complexes loaded with different crRNA1 variants (shown in B) using permanganate probing (KMnO_4_ footprinting). Lanes 3–10, probing of the complexes containing the indicated crRNA1 variant. The g8 dsDNA substrate was 5′-[^32^P]-labeled on the displaced strand, and the formation of R-loop is indicated by the detection of KMnO_4_-sensitive thymine bases (shown on the image). Lane 1, intact g8 DNA treated with KMnO_4_ (no Cascade); lane 2, A+G sequencing reaction.

Previous studies have revealed the important role of PAM and crRNA spacer seed sequences in DNA target recognition by *E. coli* Cascade-crRNA complex (13,14,44,47,48). However, the importance of the crRNA handle sequences and 2′, 3′-cyclic phosphate group at the 3′-terminus of the mature crRNA is still unclear. Recent crystal structures of the *E. coli* Cascade-crRNA complex revealed that the 5′ and 3′ handles of the crRNA are anchored at the opposite ends of the complex, with the 5′-handle bound to Cas5 (CasD), whereas the 3′ handle with a hairpin is attached to the Cas6e (CasE) ribonuclease subunit (23–25). Crystal structures of the homologous Csy4 and Cse3 endoribonucleases from *Pseudomonas aeruginosa* and *T. thermophilus* in complex with crRNA confirmed that the crRNA stem-loop structure is important for the proper positioning of the pre-crRNA substrate for cleavage, which occurs at the stem base (32,49). To characterize the role of the crRNA spacer flanking sequences in the binding of a mature crRNA to Cascade and formation of the R-loop, we prepared a series of synthetic crRNA variants (crRNA2 to crRNA7) trimmed either from the 5′- or 3′-end and analyzed their binding to the purified Cascade^−^ and binding of the respective Cascade-crRNA complexes to the g8 target dsDNA (Figure 5A, Supplementary Figure S2). The removal of the 2′, 3′-cyclic phosphate from the 3′-terminus of crRNA1 (in crRNA2, −8/+21) had no effect on its binding to Cascade^−^, and the binding affinity of the Cascade-crRNA2 complex to the g8 dsDNA was close to that of the Cascade-crRNA1 complex (Figure 5, Supplementary Figure S2). In contrast, deletion of 5 nt of the 5′ handle (in crRNA3, −3/+21) reduced its binding to Cascade^−^, whereas an even stronger negative effect was observed after the deletion of 8 nt (in crRNA4, −0/+21) (Figure 5). The Cascade complexes loaded with crRNA3 and crRNA4 also showed greatly reduced DNA target recognition (Figure 5, Supplementary Figure S2). Similarly, deletion of 6 nt from the 3′-end (in crRNA5, −8/+15) or complete removal of the 3′ handle (in crRNA6, −8/+0) had a strong negative effect on crRNA binding to Cascade^−^ and on the g8 dsDNA binding by the respective Cascade complexes (Figure 5). Unexpectedly, the deletion of 16 nt from the 3′-end of crRNA1 leaving just five nucleotides of the 3′ handle (crRNA7, −8/+5), allowed binding to Cascade^−^ and g8 dsDNA target recognition with binding affinities just slightly lower than those of crRNA1 (Figure 5, Supplementary Figure S2). The permanganate probing analysis confirmed the formation of the R-loop by the Cascade^−^ complexes loaded with crRNA2 and crRNA7, whereas no R-loop formation was detected with crRNA3-crRNA6 (Figure 5C). Additional binding experiments using crRNAs containing longer (crRNA11, −8/+18) or shorter (crRNA12, −8/+11; crRNA13, −8/+9) 3′ handles showed detectable binding to Cascade^−^ (Supplementary Figure S3). Bioinformatic analysis of potential secondary structures of crRNA5, 7, 11, 12 and 13 revealed that the inactive crRNA5 can form structures with the lowest predicted free energy compared to other crRNAs (Supplementary Figure S3) suggesting that these stable secondary structures can prevent specific binding of crRNA5 to Cascade. Be that as it may, our results indicate that the 5′ handle of the *E. coli* crRNA is crucial for its binding to Cascade and subsequent DNA target recognition, whereas just five nucleotides of the 3′-handle are sufficient for crRNA function.

**Figure 6.**
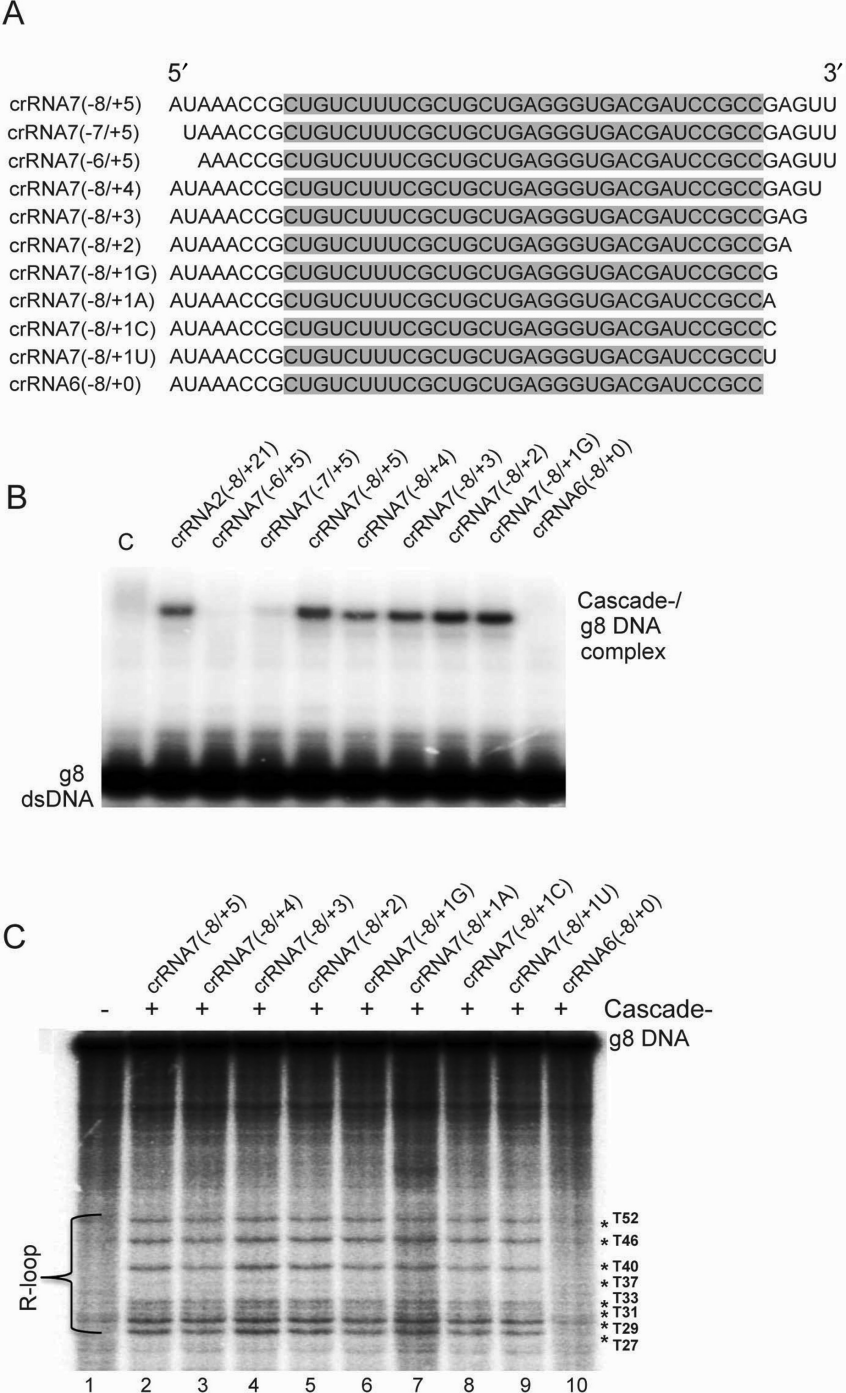
DNA target recognition and R-loop formation by Cascade complexes loaded with different crRNAs. (**A**) Sequences of the crRNA variants with different nucleotide deletions or substitutions in the 5′ or 3′ handle used in these experiments. The g8 spacer sequence is shaded in gray. (**B**) DNA targeting: g8 dsDNA binding by the Cascade^−^ complexes loaded with the indicated crRNA variant (analyzed by native gel electrophoresis as in Figure 4). (**C**) R-loop formation: permanganate probing of the Cascade^−^/crRNA/g8 dsDNA complexes shown in panel B. Lane 1, the 5′- [^32^P]-labeled g8 DNA substrate incubated with KMnO_4_ (without Cascade); lanes 2–10, probing of R-loop formation by the Cascade complexes loaded with the indicated crRNA variants (analyzed by denaturing PAGE as in Figure 4).

In the second series of trimmed crRNAs, we removed single nucleotides from both 5′ and 3′ handles of crRNA7 (−8/+5) and analyzed the DNA target recognition and R-loop formation activities of the Cascade^−^ complexes loaded with these crRNA derivatives. We found that the removal of one or two nucleotides from the 5′ handle abolished the dsDNA target recognition indicating that all eight nucleotides of the 5′ handle are critical for Cascade activity (Figure 6). Recent crystal structures of the *E. coli* Cascade-crRNA complexes revealed that the crRNA 5′ handle is buried between Cas5, Cas7.6 and Cse1 forming a hook-like structure with extensive contacts with these Cascade subunits including several base-specific interactions (23–25). In contrast, successive (one by one) trimming of the crRNA7 3′ handle produced several active crRNAs. Only the total deletion of the 3′ handle resulted in complete inhibition of dsDNA target recognition (Figure 6). These experiments reveal that just one nucleotide flanking the crRNA spacer at the 3′-end is sufficient for dsDNA target recognition and R-loop formation. Interestingly, the type of base of the first 3′ handle nucleotide (+1) was not important for binding since, in addition to G encoded by the *E. coli* repeat, crRNA7 variants with A, C or U in this position also bound Cascade forming complexes capable of target recognition (Figure 6). Based on the available crystal structure of the *E. coli* Cascade-crRNA complex (23), the +1 nucleotide of the crRNA 3′ handle can interact with the Cascade Cas7.1, Cas7.2 and Cse2.1 subunits suggesting that these interactions are important for crRNA binding. Thus, all eight nucleotides of the crRNA 5′ handle are required for dsDNA target recognition and R-loop formation, whereas just one 3′ handle nucleotide is sufficient for these activities *in vitro*.

**Figure 7.**
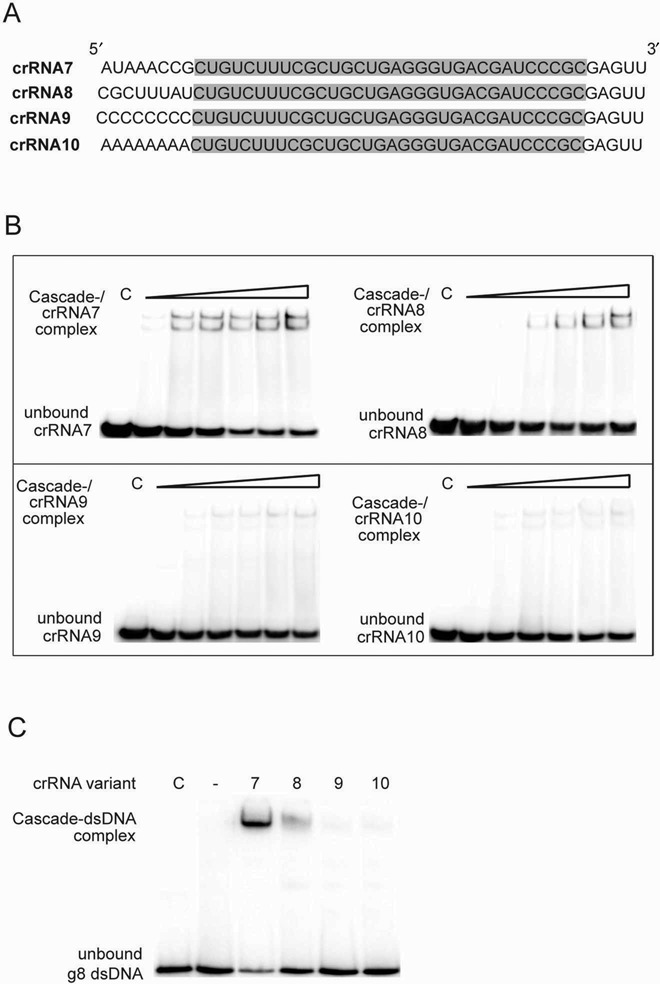
Effect of the crRNA 5′ handle sequence on its binding to Cascade^−^ and dsDNA targeting. (**A**) crRNA7 and its variants with different 5′ handle sequences. The g8 spacer is shaded in gray. (**B**) EMSA assays showing binding of the crRNA7 variants to Cascade^−^. The indicated 5′-[^32^P]-labeled crRNAs (10 nM) were incubated with purified Cascade^−^ (0–500 nM) at 37°C for 5 min, resolved on 6% native polyacrylamide gels, and visualized by phosphorimaging. (**C**) Binding of Cascade^−^ loaded with the indicated crRNA variants to the g8 dsDNA. Purified Cascade^−^ (500 nM) was loaded with the indicated non-labeled crRNA (10 nM, 10 min at 30°C), then the 5′-[^32^P]-labeled g8 dsDNA (10 nM) was added and incubated for 30 min at 37°C. Reaction products were resolved by native PAGE (6%) and visualized by phosphorimaging.

The higher affinity of Cascade^−^ to crRNA and the dispensability of most of its 3′-handle for binding to Cascade^−^ suggest that the crRNA 5′-handle might be responsible for the specific recognition of crRNA by a Cascade complex. This is consistent with the recent crystal structures of the *E. coli* Cascade-crRNA complex, which revealed several sequence-specific interactions between the crRNA 5′ handle (−7, −5, −3 and −2) and the Cas5 and Cas7.6 subunits (23–25). To confirm this, we analyzed binding of Cascade^−^ to the three crRNA7 variants (45 nt) with different 5′-handle 8 nt sequences. As shown in Figure 7, the Cascade^−^ showed a greatly reduced binding to the crRNA7 variants containing the poly-C and poly-A 5′-handles (crRNA8 and crRNA9), but detectable binding to crRNA10 with the 5′-handle containing a random sequence. DNA target recognition experiments using these crRNAs and Cascade^−^ revealed no binding to the g8 ssDNA in the presence of crRNA8 (polyC 5′ handle) and crRNA9 (polyA 5′ handle) (Figure 7). The crRNA10 with a random sequence in the 5′ handle supported weak DNA binding by Cascade^−^, which was significantly lower than in the presence of crRNA7 (Figure 7). These results indicate that both the length and sequence of the crRNA 5′ handle are important for Cascade binding to crRNA and for DNA target recognition.

## DISCUSSION

Recognition of foreign DNA in type I and type III CRISPR-Cas systems is carried out by multi-subunit ribonucleoprotein effector complexes, which are called Cascade (*C*RISPR-*as*sociated *c*omplex for *a*ntiviral *de*fense) complexes in the case of the *E. coli* type I-E system. For *E. coli* Cascade and other effector complexes where Cas6 is an integral subunit, it is generally accepted that ribonucleoprotein assembly is initiated by site-specific cleavages of the CRISPR array transcript by Cas6, which then remains tightly bound to the mature crRNA formed and promotes the assembly of other Cascade subunits (18,23,49,50). There is also the alternative possibility that Cas6 releases mature crRNAs, which then associate with preformed Cascade complexes and/or Cascade subunits (34). The data presented here indicate that a functionally active *E. coli* Cascade complex (Cascade^−^) can be purified from *E. coli* cells lacking crRNA. These results suggest that Cascade complexes might also assemble in the absence of crRNAs or using non-crRNAs as a scaffold, which can then be replaced by mature crRNAs. Purified *E. coli* Cascade^−^ can be loaded *in vitro* with different crRNAs, which direct it to complementary DNA targets (Figure 3). The functionality of the *in vitro* loaded Cascade-crRNA complex is demonstrated by the formation of an R-loop on dsDNA targets and by the inhibiting effect of an escape mutation in the DNA target protospacer (Figures 3 and 4).

The availability of *in vitro* reconstituted Cascade-crRNA complexes allowed us to address structure–function relationships in crRNA that could not have been addressed before. We used purified Cascade^−^ complex to analyze the importance of crRNA 5′ and 3′ handles for Cascade activity. We found that all eight nucleotides of the crRNA 5′ handle are required for crRNA binding to Cascade^−^ and DNA target recognition as deletion of even a single nucleotide produced a strong negative effect. In addition to the length of the crRNA 5′ handle, its sequence also plays an important role in binding to Cascade. This is consistent with the sequence-specific recognition of the crRNA 5′ handle by the Cascade subunits shown by the recent crystal structures of the *E. coli* Cascade-crRNA complexes (23–25). These structures revealed that all eight nucleotides of the 5′ handle are buried in a pocket formed by Cas5, Cas7.6 and Cse1 with many specific interactions between the nucleotides and amino acid residues. It is interesting that most of the characterized CRISPR effector complexes (Cascade, CMR, CSM) use crRNAs with an 8-nt 5′ handle (5,33,50,51). In the complex CRISPR system of *S. solfataricus*, the mature crRNAs produced from the six CRISPR loci have identical 5′ handles, which can be used by the three different effector complexes (Cascade I-A, CSM and CMR) (30,33,34). Thus, the 8-nt 5′ handle of crRNA plays a crucial role in the function of the three major CRISPR effector complexes including Cascade, CMR and CSM.

In contrast, most of the 21 nt of the 3′ handle of the *E. coli* crRNA were dispensable for its binding to Cascade with just one nucleotide flanking the spacer sequence at the 3′-end sufficient for Cascade binding and DNA target recognition. This is consistent with the previous analysis of crRNAs extracted from the *E. coli* Cascade, which revealed that all crRNAs contained the full-length (8 nt) 5′ handle followed by a complete spacer sequence (32 nt) and a 3′ handle of varying length (8–19 nt) (7). Similarly, the analysis of crRNAs extracted from the *S. solfataricus* Cascade and type III complexes (CSM and CMR) demonstrated the presence of the 8-nt long 5′ handle with varying 3′ ends (26,30,33). Based on the crystal structures of the *E. coli* Cascade-crRNA complexes (23–25), the significance of the first nucleotide of the 3′ handle (+1G) might be linked to its interactions with the residues of Cas7.1, Cas7.2 and Cse2.1. In addition, these structures revealed that most of the 3′ handle nucleotides including its stem-loop are bound to the Cascade surface and interact mostly with the Cas6 residues.

Thus, based on this work the minimal length of the *E. coli* crRNA still active in Cascade binding and DNA target recognition can be reduced from 61 nt found in major species of natural crRNA to 41 nt. The type II CRISPR system of *Streptococcus pyogenes* employs the programmable dual-RNA-guided Cas9 protein as an effector nuclease, which needs both a crRNA (39–42 nt) and a trans-encoded small RNA (tracrRNA, 75 nt) for DNA targeting (52). Recently, this dual crRNA system was adapted to function as a single RNA-guided Cas9 nuclease using a shorter chimeric guide RNA (62 nt), which established an efficient RNA programmable technology for gene targeting and genome editing (8). Our work together with previous studies highlights the flexibility of the Cascade-crRNA system suggesting that novel RNA-guided molecular tools can be engineered for applications in genome regulation and editing. In addition, *in vitro* reconstitution of functional Cascade-crRNA complexes allows incorporation of specific labels in defined positions of crRNA, which should open the way for powerful biochemical and biophysical analyses of Cascade-crRNA interactions with its targets.

## SUPPLEMENTARY DATA

Supplementary Data are available at NAR Online.

SUPPLEMENTARY DATA
