# Supplementary material for: CRISPR RNA binding and DNA target recognition by purified Cascade complexes from Escherichia coli
Source: Nucleic Acids Res. 2014 Dec 8;43(1):530–43. doi: 10.1093/nar/gku1285 (PMC4288178; doi:10.1093/nar/gku1285)
Supplement: SUPPLEMENTARY DATA [file supp_43_1_530__index.html]

CRISPR RNA binding and DNA target recognition by purified Cascade complexes from Escherichia coli — SUPPLEMENTARY DATA 

# CRISPR RNA binding and DNA target recognition by purified Cascade complexes from *Escherichia coli*

## SUPPLEMENTARY DATA

**Files in this Data Supplement:**

- SUPPLEMENTARY DATA
