# Supplementary material for: CRISPR RNA binding and DNA target recognition by purified Cascade complexes from Escherichia coli
Source: Nucleic Acids Res. 2014 Dec 8;43(1):530–43. doi: 10.1093/nar/gku1285 (PMC4288178; doi:10.1093/nar/gku1285)
Supplement: SUPPLEMENTARY DATA [file supp_gku1285_nar-02573-h-2014-File009.pdf]

Supplementary Materials

CRISPR RNA binding and DNA targeting by purified Cascade complexes from *Escherichia coli*

Natalia Beloglazova[1*], Konstantin Kuznedelov[2*], Robert Flick[1], Kirill A. Datsenko[3], Greg Brown[1], Ana Popovic[1], Sofia Lemak[1], Ekaterina Semenova[2], Konstantin Severinov[2#], and Alexander F. Yakunin[1#]

Supplementary Figure Legends

Supplementary Figure S1. Cascade purification and analysis. (**A**), Size-exclusion elution profiles (Superdex 200 HR 10/30 (GE)) of the purified Cascade^{+} and Cascade^{-} complexes; (**B**), Denaturing gels showing [^{32}P]labelled nucleic acids phenol-extracted from the complexes Cascade + and Cascade- (lanes 1,2) and nucleic acids treatment with DNaseI (lanes 3,4) or RNaseA (lanes 5, 6). Lane M - molecular size marker, A260/A280 ratio is shown. (**C**), Binding curves for the purified Cascade^{+} and Cascade^{-} and different 5′-[^{32}P]-labelled RNAs and DNAs, quantitative analysis of EMSA gels shown on Figure 2; (**D**), EMSA showing binding of 3$^{/}$ -[^{32}P]-end labelled crRNA7 to Cascade^{-} complex. (**E**), EMSA and permanganate probing of Cascade^{+} and Cascade^{-}. Cascade^{+} was purified from the *E. coli* KD418 strain co-transformed with a modified pWUR400 (expressing the Cascade complex with the N-terminal Strep-tag fused to Cse2) and pWUR615 (expressing an engineered *E. coli* CRISPR cassette with seven g8 spacers) (Semenova et al., 2011). Cascade^{-} was purified from the *E. coli* KD418 strain expressing the Cascade complex with the N-terminal 6His-tag fused to Cse1). It was loaded with

synthetic crRNA7 before the experiments. The target g8 dsDNA (209 bp) was PCR amplified and labelled with 5′-[^{32}P]. Binding reactions were performed for 30 min at 37 °C and permanganate probing was performed as described in Materials and Methods. C, control without Cascade addition.

Supplementary Figure S2. EMSA analysis of crRNA binding. Left panels: binding of purified Cascade$^-$ to the 5′-[^{32}P]-labelled crRNA variants. Right panels: binding of Cascade$^-$ complexes loaded *in vitro* with the indicated crRNA variants to the 5′-[^{32}P]-labelled g8 dsDNA.

Supplementary Figure S3. EMSA and permanganate probing. (**A**), g8 dsDNA binding by the Cascade$^-$ complexes loaded with the indicated crRNA variant (analysed by native gel electrophoresis). (**B**), R-loop formation: permanganate probing of the Cascade$^-$/crRNA/g8 dsDNA complexes shown in panel A. Lanes 1,8, the [5'- ^{32}P]-labelled g8 DNA substrate incubated with $KMnO_4$ (without Cascade); lanes 2-7, probing of R-loop formation by the Cascade complexes loaded with the indicated crRNA variants (analyzed by denaturing PAGE. (**C**) Secondary structures of indicated crRNA predicted using Mfold software.

Supplementary Figure S1

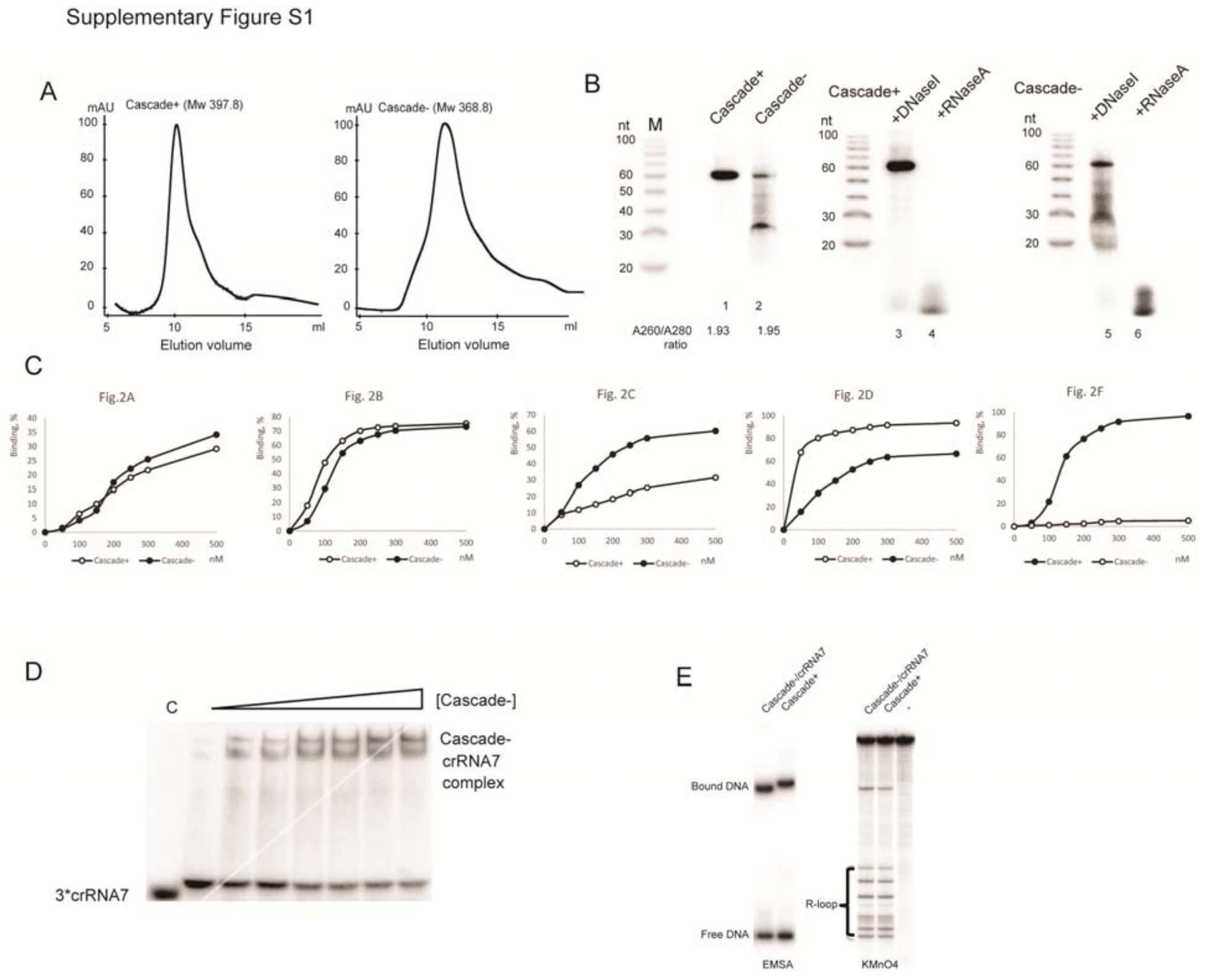

Supplementary Figure S2

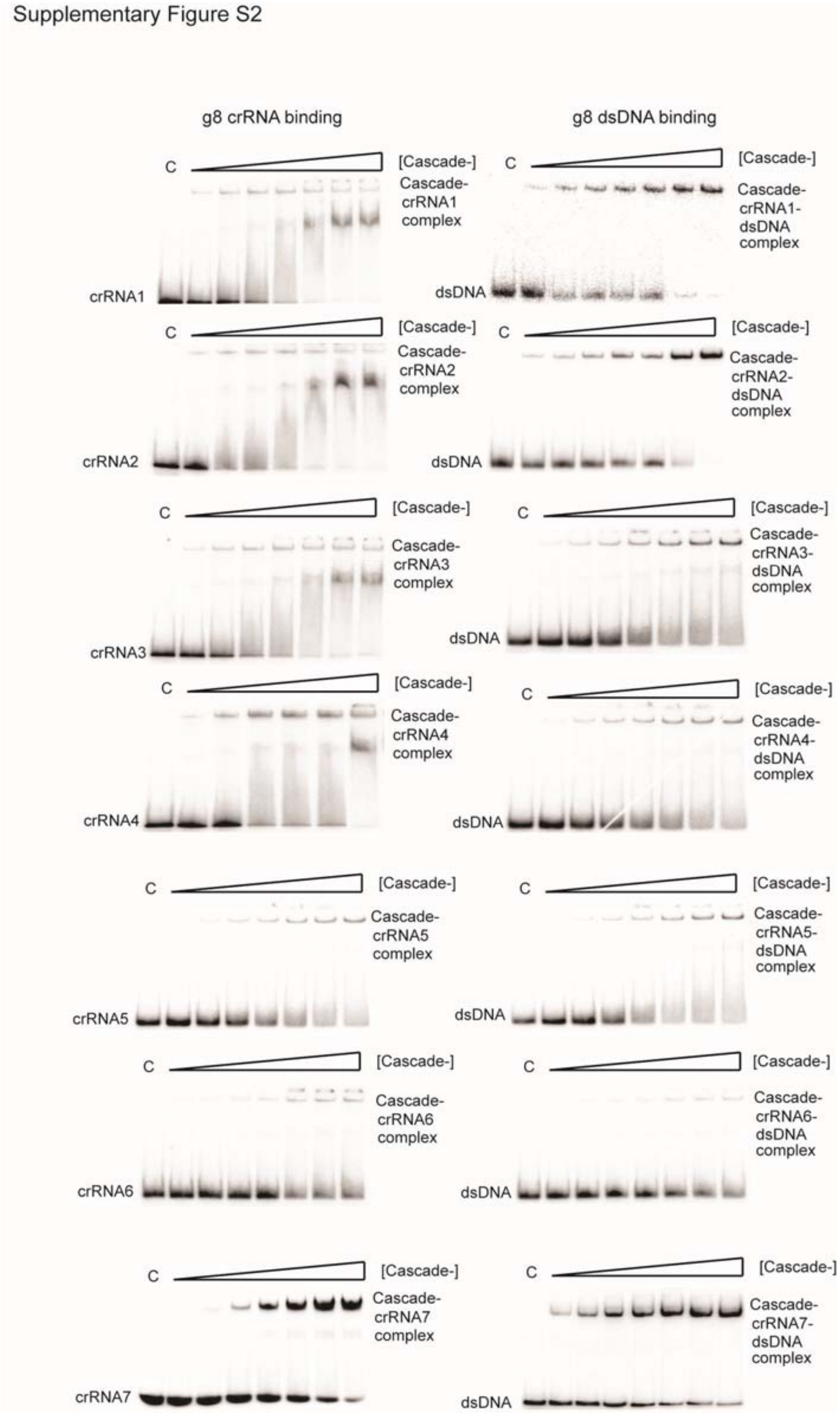

Supplementary Figure S3

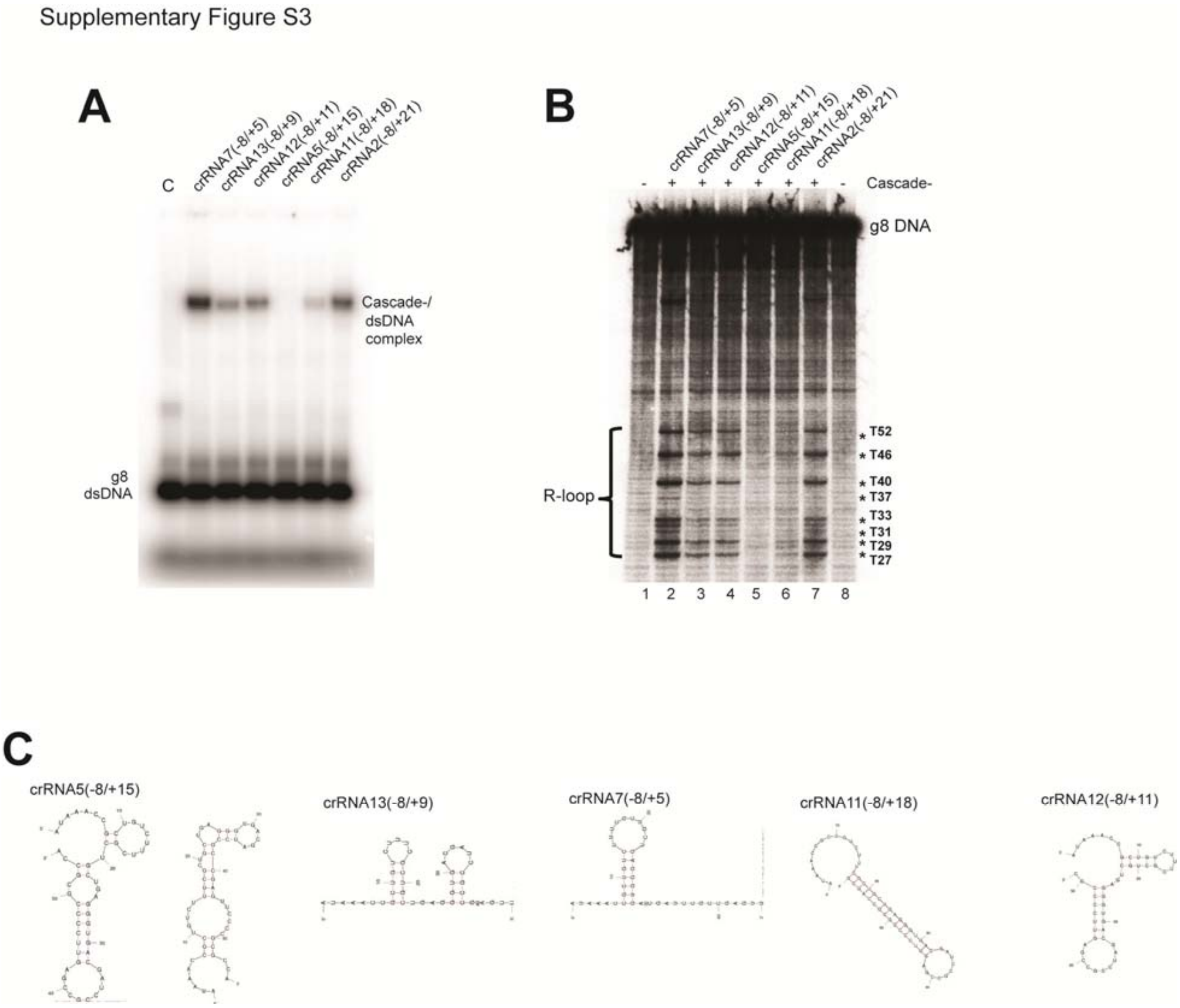

Supplementary Table S1. Oligonucleotides used for the preparation of DNA and RNA substrates

Substrate	Length (nt)	Sequence (5′-3′)
1. ssRNA	61	CACUGUGAUGACGAAGCUUGCGGCCGCACUCGAGGAUCCGGCUGCACAAAGCCCGAAAGU
2. crRNA1(-8/+21)	61	AUAAACCGCUGUCUUUCGCUGCUGAGGGUGACGAUCCGCCGAGUUCCCCGCGCCAGCGGGG
3. crRNA2(-8/+21)	61	AUAAACCGCUGUCUUUCGCUGCUGAGGGUGACGAUCCGCCGAGUUCCCCGCGCCAGCGGGG
4. crRNA3(-3/+21)	56	CCGCUGUCUUUCGCUGCUGAGGGUGACGAUCCGCCGAGUUCCCCGCGCCAGCGGGG
5. crRNA4(-1/+21)	54	GCUGUCUUUCGCUGCUGAGGGUGACGAUCCGCCGAGUUCCCCGCGCCAGCGGGG
6. crRNA5(-8/+15)	55	AUAAACCGCUGUCUUUCGCUGCUGAGGGUGACGAUCCGCCGAGUUCCCCGCGCCA
7. crRNA6(-8/+0)	40	AUAAACCGCUGUCUUUCGCUGCUGAGGGUGACGAUCCGCC
8. crRNA7(-8/+5)	45	AUAAACCGCUGUCUUUCGCUGCUGAGGGUGACGAUCCGCCGAGUU
9. crRNA7(-7/+5)	44	UAAACCGCUGUCUUUCGCUGCUGAGGGUGACGAUCCGCCGAGUU
10. crRNA7(-6/+5)	43	AAACCGCUGUCUUUCGCUGCUGAGGGUGACGAUCCGCCGAGUU
11. crRNA7(-8/+4)	44	AUAAACCGCUGUCUUUCGCUGCUGAGGGUGACGAUCCGCCGAGU
12. crRNA7(-8/+3)	43	AUAAACCGCUGUCUUUCGCUGCUGAGGGUGACGAUCCGCCGAG
13. crRNA7(-8/+2)	42	AUAAACCGCUGUCUUUCGCUGCUGAGGGUGACGAUCCGCCGA
14. crRNA7(-8/+1G)	41	AUAAACCGCUGUCUUUCGCUGCUGAGGGUGACGAUCCGCCG
15. crRNA7(-8/+1A)	41	AUAAACCGCUGUCUUUCGCUGCUGAGGGUGACGAUCCGCCA
16. crRNA7(-8/+1C)	41	AUAAACCGCUGUCUUUCGCUGCUGAGGGUGACGAUCCGCCC
17. crRNA7(-8/+1U)	41	AUAAACCGCUGUCUUUCGCUGCUGAGGGUGACGAUCCGCCU
18. crRNA8(-8/+5)	45	CGCUUUAUCUGUCUUUCGCUGCUGAGGGUGACGAUCCGCCGAGUU
19. crRNA9(-8/+5)	45	AAAAAAAACUGUCUUUCGCUGCUGAGGGUGACGAUCCGCCGAGUU
20. crRNA10(-8/+5)	45	CCCCCCCCCUGUCUUUCGCUGCUGAGGGUGACGAUCCGCCGAGUU
21. crRNA11(-8/+18)	58	AUAAACCGCUGUCUUUCGCUGCUGAGGGUGACGAUCCGCCGAGUUCCCCGCGCCAGCG
22. crRNA12(-8/+11)	51	AUAAACCGCUGUCUUUCGCUGCUGAGGGUGACGAUCCGCCGAGUUCCCCGC
23. crRNA13(-8/+9)	49	AUAAACCGCUGUCUUUCGCUGCUGAGGGUGACGAUCCGCCGAGUUCCCC
24. crRNA MS2(-8/+21)	61	AUAAACCGGCACCGACCCCUUUCUGGAGGUACAUAUUCAUGAGUUCCCCGCGCCAGCGGGG
25. DNA1	79	CAGGGAGTTAAAGGCCGCTTTTGCGGGATCGTCACCCTCAGCAGCGAAAGACAGCATCGGAACGAGGGTAGCAACGGCT
26. DNA2	79	AGCCGTTGCTACCCTCGTTCCGATGCTGTCTTTCGCTGCTGAGGGTGACGATCCCGCAAAAGCGGCCTTTAACTCCCTG
27. DNA3	79	CAGGGAGTTAAAGGCCGCTTTTGCGGGATCGTCACCCTCAGCAGCGAAAGACAACATCGGAACGAGGGTAGCAACGGCT
28. DNA4	79	AGCCGTTGCTACCCTCGTTCCGATGTTGTCTTTCGCTGCTGAGGGTGACGATCCCGCAAAAGCGGCCTTTAACTCCCTG
29. DNA5	80	TAGAGTCTTATATGAGAAAAAAAGTTAAAATTTTTTATTAGCTTTCACGATAACGATTACAACAGTTATTTGGTAAGAGC
30. DNA6	80	GCTCTTACCAAATAACTGTTGTAATCGTTATCGTGAAAGCTAATAAAAAATTTTAACTTTTTTTCTCATATAAGACTCTA
31. DNA7	72	TGAGCCGGCGTCTGATAAAGGCACCGACCCCTTTCTGGAGGTACATATTCATATCAGGCTCCTTACAGGCAG
32. DNA8	72	CTGCCTGTAAGGAGCCTGATATGAATATGTACCTCCAGAAAGGGGTCGGTGCCTTTATCAGACGCCGGCTCA

Underlined nucleotides in crRNAs represent a spacer sequence.

Supplementary Table S2. Identification of Cas proteins by tryptic digest LC-MS

Sample	Protein Identity	log(e)	log(I)	Protein Coverage		Unique Peptides	Total Peptides	Molecular Weight (kDa)
				% measured	% corrected			
**SDS-PAGE of Cascade$^-$ (after affinity purification)**								
Band-1 (Cse1)	Cse1	-357.7	8.9	68	100+	40	106	58.3
Band-2 (Cas7)	Cas7	-388.8	9.38	72	100+	43	176	40
	Cse1	-57	7.47	15	26	8	16	58.3
Band-3 (Cas5)	Cas5	-148.6	8.53	56	62	19	64	25.2
	Cse1	-68.2	7.19	15	26	10	14	58.3
Band-4 (Cas6e)	Cas6e	-47.3	8.3	27	35	7	28	22.3
	Cas5	-40.9	7.49	29	32	6	12	25.2
Band-5 (Cse2)	Cse2	-155.5	9	74	94	20	91	18.7
**Size-exclusion chromatography samples (in solution)**								
Cascade$^+$	Cse1	-1462.4	8.48	73	100+	111	1527	58.3
	Cas7	-424.1	7.16	64	98	30	202	40
	Cas5	-139.6	6.41	44	49	12	31	25.2
	Cse2	-103.9	6.46	53	67	9	34	18.7
	Cas6e	-23.8	5.42	18	23	3	8	22.3
Cascade$^-$	Cse1	-2202.8	8.94	84	100+	166	2660	58.3
	Cas7	-337	7.16	57	88	23	128	40
	Cas5	-102.1	6.79	40	45	10	31	25.2
	Cse2	-89.5	6.36	45	57	8	24	18.7
	Cas6e	-23.4	6.1	15	19	3	8	22.3

log(e): base-10 log of the expectation that any particular protein assignment was made at random (E-value)
log(I): base-10 log of the sum of fragment ion intensities used for protein assignment
% measured: amino acid coverage of assigned protein
% corrected: amino acid coverage of assigned protein, corrected for peptides unlikely to be observed
Unique Peptides: Number of unique peptide sequences associated with protein assignment
Total Peptides: Total number of peptides used for protein assignment
